# Failure rates increase over time and are higher following medial arthroscopic bucket‐handle meniscal repair: A systematic review and meta‐analysis

**DOI:** 10.1002/jeo2.70854

**Published:** 2026-08-27

**Authors:** Amirali Soleymani Nejad, Sohrab Keyhani, Mehran Soleymanha, Fardis Vosoughi, Robert F. LaPrade, Maryam Mousavi, Luke V. Tollefson

**Affiliations:** ^1^ Poursina Hospital Orthopedic Research Center Guilan University of Medical Sciences Rasht Iran; ^2^ Bone, Joint, and Related Tissues Research Center, Akhtar Orthopedic Training and Research Hospital Shahid Beheshti University of Medical Sciences Tehran Iran; ^3^ Department of Orthopedics and Trauma Surgery, Shariati Hospital Tehran University of Medical Sciences Tehran Iran; ^4^ Department of Complex Knee and Sport Medicine Twin Cities Orthopedics Edina Minnesota USA; ^5^ Faculty of Sciences University of Guilan Rasht Iran; ^6^ Twin Cities Orthopedics Edina Minnesota USA

**Keywords:** arthroscopic surgery, bucket‐handle meniscal tear, failure rate, postoperative period, risk factors

## Abstract

**Purpose:**

Bucket‐handle meniscal tears (BHMTs) constitute a technically demanding form of meniscal injury. However, the incidence of repair failure and the factors influencing outcomes remain incompletely characterised. This study aimed to estimate the pooled failure rate after bucket‐handle meniscal repair (BHMR) and to identify variables associated with unsuccessful healing.

**Methods:**

A systematic review and meta‐analysis (SRMA) was performed to identify studies published from January 2005 through 2026 evaluating arthroscopic BHMR. Eligible designs included randomised trials, cohort studies and case series with at least 15 patients. Random‐effects or fixed models were used to calculate pooled failure proportions and odds ratios. Subgroup analyses and meta‐regression were conducted to explore potential predictors of failure.

**Results:**

The pooled BHMR failure rate was 21.5% (95% CI: 16.3%–27.8%; *I* ^2^ = 79.2%). Failure rates increased with longer follow‐up (*β* = 0.12, *p* < 0.01). Medial repairs had significantly higher failure odds than lateral repairs (OR = 2.20, *p* < 0.001, pooled failure rate: 18.9% vs. 12.1%). No significant differences were observed for tear zone (red‐red vs. white‐red), sex or isolated (pooled rate: 24.3%) versus concomitant ACLR (pooled rate: 14.9%). Publication bias was suggested by Egger's test (*p* = 0.02).

**Conclusion:**

BHMRs carry a substantial 21.5% failure rate that increases over time, with medial tears at significantly higher risk than lateral tears. Concomitant ACL reconstruction and tear vascularity did not significantly impact failure odds. High‐quality prospective studies are needed to optimise patient selection and long‐term counselling.

**Level of Evidence:**

Level II.

**Abbreviations**
ACLR: anterior cruciate ligament reconstruction
BHMR: bucket‐handle meniscal repair
BHMTs: bucket‐handle meniscal tears
CI: confidence interval
IQR: interquartile range
OR: odds ratio
PRISMA: preferred reporting items for systematic reviews and meta‐analyses
RCT: randomised controlled trial
SRMA: systematic review and meta‐analysis

## INTRODUCTION

Meniscal injuries are among the most common intraarticular knee injuries account for approximately 14.5% of all athletic knee injuries [51]. The meniscus is essential for several aspects of knee function, such as proprioception, lubrication, joint stability, load distribution and shock absorption [5, 54, 75, 88, 95]. Many authors support meniscal preservation through repair because it may postpone the development of degenerative joint changes [68]. Therefore, wherever possible, meniscal repair is preferred to meniscectomy in modern surgical practice [41].

Bucket‐handle meniscal tears (BHMT), which account for approximately 10%–26% of lesions, usually involve a displaced longitudinal tear extending into the intercondylar notch or along the femoral condyle [20, 52, 76, 90]. Therefore, pain, perceived instability and mechanical locking are typical clinical signs [35, 69, 70, 76]. The prevalence of BHMT across 1485 isolated meniscal tears of stable knees was reported 8.7%; however, it was higher in male patients (11%) under 40 [56]. Furthermore, prior research has shown a high rate of BHMT in conjunction with anterior cruciate ligament (ACL) injuries [40, 92].

Arthroscopic repair techniques include outside‐in, inside‐out, all‐inside or the hybrid approach that combines these techniques [11]. When the posterior portion of the medial meniscus is involved, the inside‐out repair approach poses the risk of neurovascular injury. To lower the potential of neurovascular injury, an all‐inside healing procedure was introduced [3, 44].

In 2021, Costa et al. published a systematic review and meta‐analysis (SRMA) evaluating failure rates following bucket‐handle meniscal repair (BHMR) [18]. Since then, the available evidence base has expanded considerably, with the publication of several large cohort studies, studies reporting longer‐term follow‐up and investigations exploring factors associated with repair failure. Despite these advances, uncertainty remains regarding the true pooled failure rate after arthroscopic BHMR and the influence of key clinical variables. Therefore, an updated SRMA were undertaken to determine the pooled failure rate following arthroscopic BHMR. Secondary objectives were to identify factors associated with repair failure through meta‐regression analyses and to compare outcomes across clinically relevant subgroups.

## MATERIALS AND METHODS

This review was performed in compliance with the preferred reporting items for systematic reviews and meta‐analyses (PRISMA) 2020 statement [63]. A protocol for this review was prospectively registered with PROSPERO before the conduct of the final analysis. The literature search was conducted on 8 June 2026, across multiple databases, including PubMed, Scopus, Embase, the Cochrane Library and Web of Science. The full search strategy is provided in the Supplementary Material (Supporting Information: Table S1).

### Eligibility criteria

Studies were eligible if they involved human subjects undergoing arthroscopic repair of medial and/or lateral BHMTs and reported repair failure rates or provided sufficient data for the extraction of BHMT‐specific failure outcomes. Included studies were required to be published in English between January 2005 and the date of the final literature search, have a minimum follow‐up of 2 years, and include at least 15 patients. Exclusion criteria comprised case reports, reviews, editorials, commentaries, letters, conference abstracts, theses, dissertations, unpublished or non–peer‐reviewed studies, animal or laboratory investigations, cadaveric studies without clinical outcomes, studies lacking extractable BHMT‐specific failure data, non‐English publications and studies published before 2005.

### Study selection

**Figure 1:**
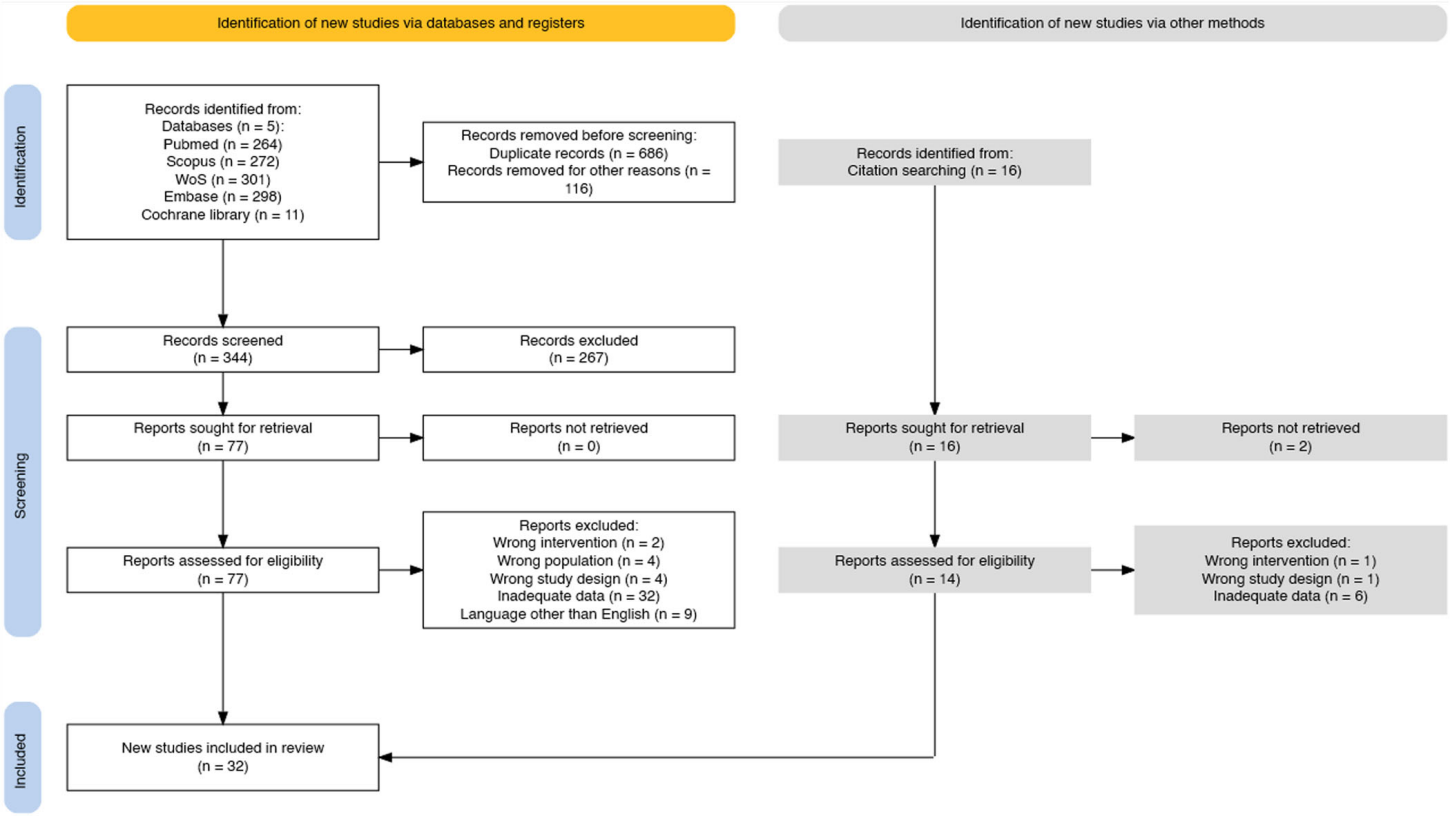
PRISMA 2020 flow diagram: Study selection process for BHMR systematic review and meta‐analysis. BHMR, bucket‐handle meniscal repair; PRISMA, preferred reporting items for systematic reviews and meta‐analyses.

Titles and abstracts of all retrieved references were independently screened by two reviewers for potential eligibility. Full texts of relevant articles were retrieved and assessed independently. Any disagreements were settled through consensus or by consulting a third reviewer. Exclusion reasons during full‐text screening were recorded and presented in the PRISMA flow diagram (Figure 1).

### Data extraction

Independent data extraction was carried out by two reviewers employing a standardised form. For each study, the following domains were systematically extracted: patient demographics, tear characteristics, concomitant injuries, surgical details and outcome metrics. Failure was defined per study‑specific criteria, which generally included clinical failure [10], re‑operation or recurrent mechanical symptoms. The corresponding authors were contacted for clarification or missing data (Supporting Information: Table S2). A consensus process was used to resolve discrepancies.

### Data synthesis and statistical analysis

Data were synthesised quantitatively where appropriate using meta‐analytic techniques [34, 51, 77, 89]. Pooled failure proportions were calculated using a random‐effects model to account for between‐study heterogeneity. Proportions were logit‐transformed before pooling and back‐transformed for presentation. The inverse‐variance method was used for weighting, and between‐study variance (*τ* ^2^) was estimated using restricted maximum likelihood (REML).

Cochran's *Q*‐test and the *I* ^2^ statistic were used to evaluate statistical heterogeneity. Significant heterogeneity was defined as *I* ^2^ > 50% and *p* < 0.10. Accordingly, random‐effects or fixed‐effect models were used for analyses with significant or nonsignificant heterogeneity, respectively. Prespecified subgroup analyses were performed according to study design (case series versus cohort studies), meniscal side (medial versus lateral) and follow‐up duration (2–5, 5–10 and >10 years). Subgroup differences were assessed using interaction tests. Comparative analyses and meta‐regression analyses were conducted to explore the association between BHMR failure rates and continuous study‐level covariates.

Sensitivity analyses were performed using leave‐one‐out analyses to evaluate the robustness of pooled estimates and to identify influential studies. All statistical analyses were conducted using R (R Foundation for Statistical Computing) 4.6.0 [67]. A two‐sided *p* < 0.05 was considered statistically significant.

### Assessment of publication bias

Publication bias was assessed visually and statistically, where sufficient studies were available. Funnel plots were generated to evaluate potential small‐study effects by plotting study‐specific effect estimates against their corresponding standard errors. Visual inspection focused on symmetry around the pooled effect estimate. In addition, a formal statistical assessment of funnel plot asymmetry was performed using Egger's regression test [22]. Because tests for publication bias have limited power when fewer than 10 studies are included, these analyses were conducted only for outcomes with an adequate number of studies [53]. Where asymmetry was observed, results were interpreted cautiously and considered exploratory.

### Risk of bias (ROB) assessment

ROB was assessed according to study design using validated, design‐specific tools. Randomised controlled trials (RCTs) were evaluated using the Cochrane Risk of Bias tool version 2.0 (RoB 2.0) [78]. Cohort studies were assessed with the Risk of Bias In Nonrandomised Studies of Interventions (ROBINS‐I) tool [77]. Case series were appraised using the Joanna Briggs Institute (JBI) Critical Appraisal Checklist for Case Series [60].

All ROB assessments were performed independently by two reviewers. Any discrepancies were resolved through discussion and consensus. For studies assessed with JBI checklists, overall methodological quality was categorised as high (7–10 domains rated as low ROB), moderate (4–6 low‐risk domains) or low (≤3 low‐risk domains). The results of the ROB assessments were summarised and visually presented using the *robvis* package in R [54].

## RESULT

### Study selection

A total of 1146 records were identified through database searching, including PubMed (*n* = 264), Scopus (*n* = 272), Web of Science (*n* = 301), Embase (*n* = 298) and the Cochrane Library (*n* = 11). After the removal of 686 duplicate records and 116 records removed for other reasons, 344 records remained for title and abstract screening. Of these, 267 records were excluded, leaving 77 reports for full‐text assessment. Additionally, 16 records were identified through citation searching, of which 14 reports were retrieved for full‐text review and 2 reports could not be obtained. Following full‐text assessment, 32 studies [4, 9, 14, 21, 24, 26, 27, 28, 29, 30, 32, 36, 37, 38, 41, 42, 46, 47, 58, 59, 60, 61, 67, 71, 72, 73, 74, 80, 82, 85, 86, 91, 93] met the predefined inclusion criteria and were included in the SRMA (Figure 1).

### Study characteristics and demographics

**Figure 2:**
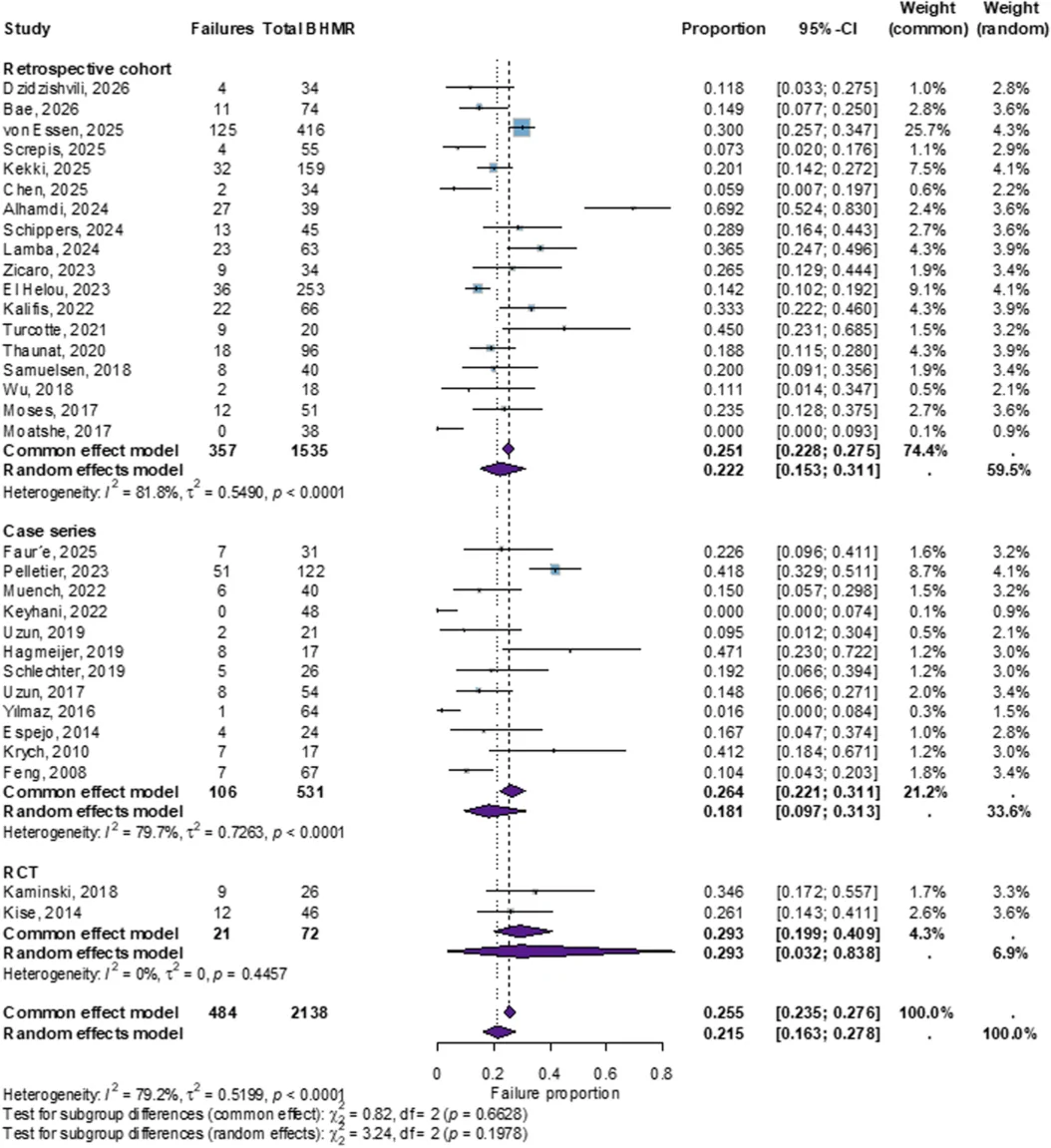
Pooled failure rate following bucket‐handle meniscal repair across all included studies, stratified by study design. CI, confidence interval; RCT, randomised controlled trial.

A total of 32 studies published between 2005 and 2026 were included in the review, comprising 2 RCTs, 18 cohort studies, 12 case‐series (Figure 2). The geographic distribution of included studies is shown in Supporting Information: Figure S1.

The sample size of BHMRs ranged from 15 to over 400 repairs, with males accounting for 66.7% of the pooled population. The mean age and mean body mass index (BMI), across studies, were 26.7 (95% confidence interval (CI): 26.2–27.2) and 24.4 (95% CI: 24.1–24.6), respectively. A detailed summary of study demographics, surgical characteristics and reported outcomes is provided in Table 1.

**Table 1:** Demographic and clinical characteristics of studies included in the systematic review and meta‐analysis of bucket‐handle meniscal repair failure.

Author, year	Country	Level of evidence	BHMR patients (*n*)	BHMR menisci (*n*)	BHMR failure (%)	Mean age (SD)	Mean BMI (SD)	Male (%)	M/L meniscus ratio	Concomitant ACLR (%)	No. of suture used mean	Zone of injury (%)			Mean F/U (SD) (years)	Suture technique (%)			
												RR	WR	WW		AI	IO	OI	Hybrid/Mixed (%)
Dzidzishvili, 2026 [21]	Italy	III	34	34	11.8	NR	NR	NR	2.4	NR	NR	NR	NR	NR	NR	NR	100.0	0.0	0.0
Bae, 2026 [9]	S. Korea	III	74	74	14.9	27.2	24.6	60	1.2	52.7	NR	50.0	50.0	0.0	5.0	8.4	0.0	0.0	0.0
von Essen et al., 2025 [26]	Sweden	III	NR	416	30.0	NR	NR	NR	2.0	0.0	NR	NR	NR	NR	NR	100.0	0.0	0.0	0.0
Screpis et al., 2025 [73]	Italy	III	55	55	7.3	NR	NR	NR	1.6	NR	NR	NR	NR	NR	NR	69.0	0.0	0.0	31.0
Kekki et al., 2025 [36]	Sweden	III	159	159	20.1	26.8 (11.2)	NR	47.8	2.8	NR	NR	NR	NR	NR	4 (NR)	0.0	0.0	0.0	100.0
Fauré et al., 2025 [27]	France	IV	31	31	22.6	13.7 (1.7)	20.4 (3.4)	83.9	0.6	0.0	4.5	NR	NR	NR	8.3 (2.9)	0.0	0.0	0.0	100.0
Chen et al., 2025 [14]	Taiwan	III	34	34	5.9	29.5 (6.5)	25.7 (4.0)	67.7	0.0	NR	NR	NR	NR	NR	4.2 (2.5)	100.0	0.0	0.0	0.0
Alhamdi et al., 2024 [4]	France	III	39	39	69.2	25.2 (6.8)	24.2 (3.7)	87.1	NR	NR	3.7	35.9	64.1	0.0	NR	0.0	0.0	0.0	100.0
Schippers et al., 2024 [71]	Germany	III	45	45	28.9	32.5 (11.2)	26.2 (6.7)	62.2	NR	NR	NR	NR	NR	NR	NR	0.0	0.0	0.0	100.0
Lamba et al., 2024 [45]	USA	III	63	63	36.5	NR	NR	NR	NR	NR	NR	NR	NR	NR	NR	41.3	58.7	0.0	0.0
Zicaro et al., 2023 [94]	Argentina	III	34	34	26.5	NR	NR	NR	NR	NR	NR	NR	NR	NR	NR	0.0	0.0	0.0	100.0
Pelletrier et al., 2023 [65]	France	IV	122	122	41.8	27.0 (9.0)	23.0 (3.0)	82.7	NR	NR	NR	44.3	47.5	8.2	8.2 (3.5)	67.2	0.0	0.0	32.8
El Helou et al., 2023 [24]	France	III	253	253	14.2	27.5 (9.0)	24.6 (3.5)	71.5	NR	100.0	3.6	NR	NR	NR	7.8 (4.0)	69.2	0.0	30.8	0.0
Muench et al., 2022 [59]	Germany	IV	40	40	15.0	32 (11.5)	25.4 (3.5)	67.5	1.5	65.0	NR	NR	NR	NR	4.3 (1.2)	0.0	0.0	0.0	100.0
Keyhani et al., 2022 [39]	Iran	IV	48	48	0.0	27 (6.8)	24.6 (2.1)	77.1	NR	NR	NR	NR	NR	NR	3.9 (0.6)	100.0	0.0	0.0	0.0
Kalifis et al., 2022 [34]	Greece	III	66	66	33.3	22.5 (5.5)	23.8 (1.7)	66.7	2.0	63.6	NR	59.0	41.0	0.0	9.6 (2.0)	0.0	0.0	0.0	100.0
Turcotte et al., 2021 [81]	USA	III	20	20	45.0	NR	NR	NR	NR	50.0	NR	NR	NR	NR	NR	100.0	0.0	0.0	0.0
Thaunat et al., 2020 [79]	France	III	96	96	18.8	27.3 (8.7)	24.8 (3.6)	72.9	NR	NR	NR	NR	NR	NR	2.9 (0.8)	0.0	0.0	0.0	100.0
Uzun et al., 2019 [85]	Turkey	IV	21	21	9.5	NR	NR	NR	0.0	47.6	3.0	NR	NR	NR	NR	0.0	0.0	0.0	100.0
Hagmeijer et al., 2019 [30]	USA	IV	17	17	47.1	16.2 (1.41)	NR	100.0	0.9	NR	NR	NR	NR	NR	18.2 (3.8)	29.4	70.6	0.0	0.0
Schlechter et al., 2019 [72]	USA	IV	26	26	19.2	NR	NR	NR	NR	NR	NR	NR	NR	NR	NR	0.0	0.0	0.0	100.0
Samuelsen et al., 2018 [70]	USA	III	40	40	20.0	NR	NR	NR	NR	62.5	NR	NR	NR	NR	4.4 (1.1)	0.0	0.0	0.0	100.0
Wu et al., 2018 [90]	USA	III	18	18	11.1	20.8 (5.1)	25.0 (3.5)	72.2	0.2	61.1	7.7	NR	NR	NR	NR	44.4	22.2	0.0	33.4
Kaminski et al., 2018 [35]	Poland	I	26	26	34.6	NR	NR	NR	NR	NR	NR	NR	NR	NR	NR	0.0	0.0	0.0	100.0
Uzun et al., 2017 [84]	Turkey	IV	54	54	14.8	NR	NR	NR	NR	NR	NR	NR	NR	NR	NR	100.0	0.0	0.0	0.0
Moses et al., 2017 [58]	USA	III	51	51	23.5	25.0 (8.7)	25.0 (4.7)	66.7	2.9	54.9	NR	NR	NR	NR	3.3 (1.4)	0.0	0.0	0.0	100.0
Moatshe et al., 2017 [57]	USA	III	38	38	0.0	31.8 (11.4)	NR	NR	NR	55.3	11.0	26.3	44.7	29.0	NR	0.0	84.2	0.0	15.8
Yılmaz et al., 2016 [93]	Turkey	IV	62	64	1.6	28.4 (7.1)	NR	58.0	2.6	62.5	NR	73.4	26.6	0.0	2.6 (0.7)	NR	NR	NR	NR
Espejo Reina et al., 2014 [25]	Spain	IV	24	24	16.7	22.8 (6.4)	NR	79.1	NR	66.7	NR	100.0	0.0	0.0	4.0 (1.9)	NR	NR	NR	NR
Kise et al., 2014 [40]	Norway	I	46	46	26.1	26.3 (4.8)	NR	56.5	22.0	10.9	NR	NR	NR	NR	NR	100.0	0.0	0.0	0.0
Krych et al., 2010 [44]	USA	IV	17	17	41.2	NR	NR	NR	NR	100.0	NR	NR	NR	NR	NR	0.0	100.0	0.0	0.0
Feng et al., 2008 [28]	China	IV	64	67	10.4	NR	NR	NR	6.4	92.5	NR	67.2	32.8	0.0	NR	4.5	35.8	0.0	59.7

Abbreviations: ACLR, anterior cruciate ligament reconstruction; AI, all‐Inside; BHMR, bucket‐handle meniscal repair; BHMT, bucket‐handle meniscal tear; BMI, body mass index; F/U, follow‐up; IO, inside‐Out; M/L, medial to lateral; NR, not reported; OI, outside‐In; RR, Red‐Red; SD, standard deviation; WR, White‐Red; WW, White‐White.

### Failure rates of BHMR

The pooled failure rate of BHMR, using a random‐effects model, was 21.5% (95% CI: 16.3%–27.8%) with substantial heterogeneity (*I* ^2^ = 79.2%, *p* < 0.001) as demonstrated in Table 2. Retrospective cohort studies demonstrated a pooled failure rate of 22.2% (95% CI: 15.3%–31.1%; *I* ^2^ = 81.8%). Sensitivity analysis showed that the pooled estimate remained stable following sequential omission of individual studies, ranging from 20.9% to 23.5% (Supporting Information: Figure S2). Case series demonstrated a pooled failure rate of 18.1% (95% CI: 9.7%–31.3%; *I* ^2^ = 79.7%). Although exclusion of Pelletier et al. [65] resulted in the greatest reduction in heterogeneity (*I* ^2^ = 66.5%), the pooled failure rate remained largely unchanged at 16.3% (95% CI: 8.6%–28.9%) (Supporting Information: Figure S2). Subgroup analysis revealed no significant differences between study designs (*p* > 0.05) (Figure 2).

**Table 2:** Summary of meta‐analyses for bucket‐handle meniscal repair failure rates.

Na	No. of studies	*I* ^2^ (%)	Model	Pooled result (95% CI) (%)	*p*‐value
Overall pooled failure rate	32	79.2	Random	21.5 (16.3–27.8)	‐
Subgroup analyses					
Mean follow‐up duration					
• 2–5 years	9	51.0	Random	18.1 (12.9–24.8)	‐
• 5–10 years	5	89.7	Random	24.1 (12.3–42.0)	‐
Study design					
• Case‐series	12	79.7	Random	18.1 (9.7–31.3)	‐
• Retrospective cohorts	18	81.8	Random	22.2 (15.3–31.1)	‐
• RCTs	2	0.0	Fixed	29.3 (3.2–83.8)	‐
Covariates					
• MBHMR	15	81.9	Random	18.9 (13.8–25.3)	‐
• LBHMR	11	0.0	Fixed	12.1 (7.9–18.1)	‐
• Isolated BHMR	10	59.9	Random	24.3 (14.3–38.2)	‐
• Concomitant ACLR BHMR	10	62.3	Random	14.9 (8.6–24.4)	‐
Comparison of covariates					
Medial versus lateral	9	0.0	Fixed	OR 2.24 (1.50–3.36)	<0.01
White–Red versus Red–Red	3	76.5	Random	OR 0.15 (0.02–1.38)	0.10
Isolated versus Concomitant ACLR BHMR	9	0.0	Fixed	OR 2.20 (1.47–3.30)	<0.01
Female versus male	3	36.8	Fixed	OR 0.96 (0.44–2.06)	0.91

*Note*: Heterogeneity was assessed using the *I* ^2^ statistic, with *I* ^2^ > 50% indicating substantial heterogeneity. Model selection (fixed‐effects or random‐effects) was based on the presence and degree of statistical heterogeneity.

Abbreviations: ACLR, anterior cruciate ligament reconstruction; BHMR, bucket‐handle meniscal repair; CI, confidence interval; LBHMR, lateral bucket‐handle meniscal repair; MBHMR, medial bucket‐handle meniscal repair; OR, odds ratio; RCT, randomised controlled trial; *I* ^2^, Higgins' heterogeneity statistic.

**Figure 3:**
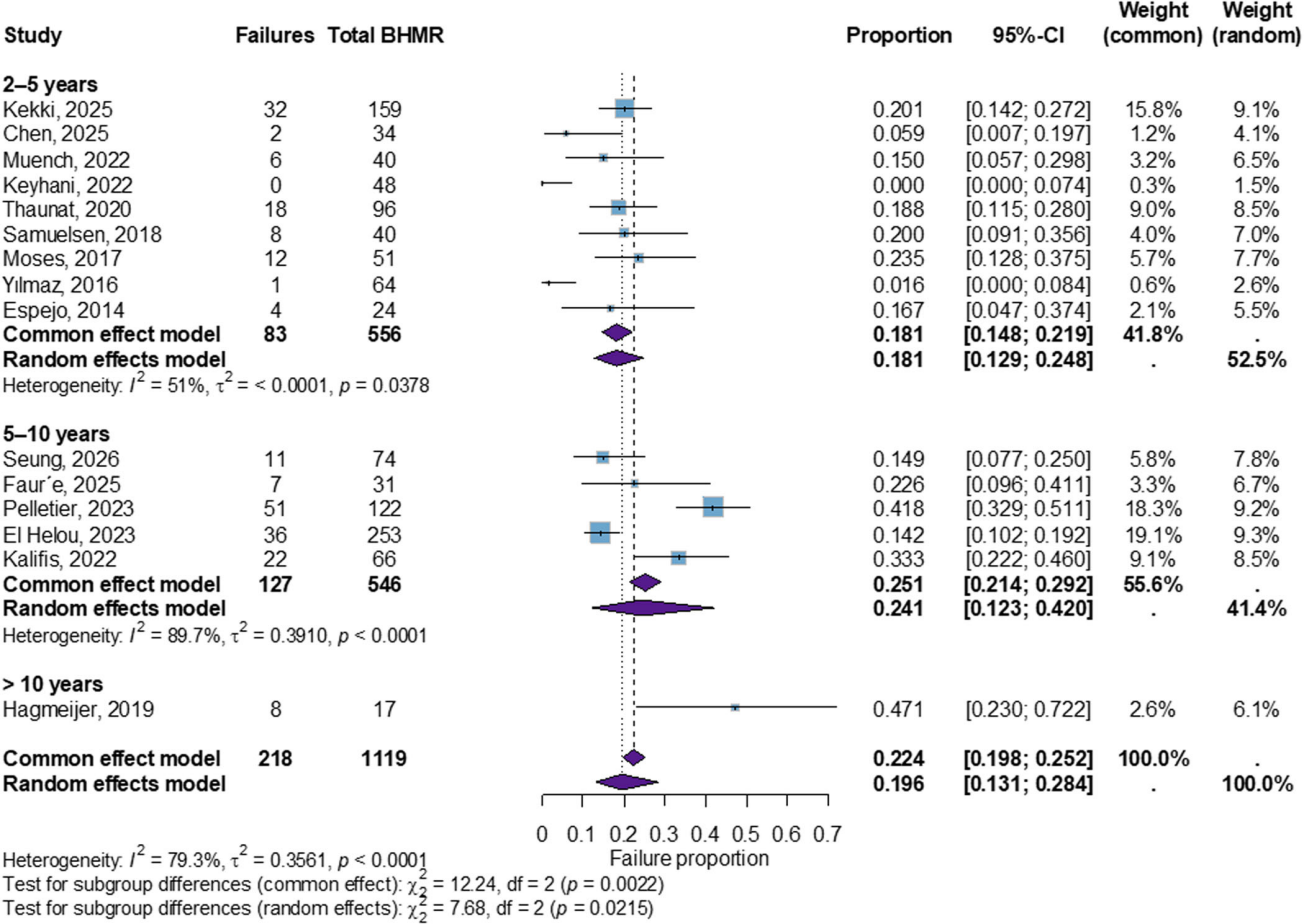
Forest plot of failure proportions for BHMR, stratified by duration of follow‐up. CI, confidence interval; BHMR, bucket‐handle meniscal repair.

As demonstrated in Figure 3, failure rates demonstrated a progressive, monotonic increase with longer follow‐up duration. Studies in the 2–5 years cohort reported a failure rate of 18.1% (95% CI: 12.9%–24.8%). Thereafter, failure rates increased substantially to 24.1% (95% CI: 12.3%–42.0%) in studies with 5–10 years of follow‐up. The pooled failure rates for medial BHMRs, lateral BHMRs, isolated BHMRs and BHMRs performed concomitantly with ACL reconstruction were 18.9% (95% CI, 13.8%–25.3%), 12.1% (95% CI, 7.4%–19.3%), 24.3% (95% CI, 14.3%–38.2%) and 14.9% (95% CI, 8.6%–24.4%), respectively (Supporting Information: Figures S3 and S4). Heterogeneity varied considerably across subgroups (*I* ^2^ range = 51.0%–89.7%) as presented in Table 2.

### Subgroup analyses of factors associated with BHMR failure

**Figure 4:**
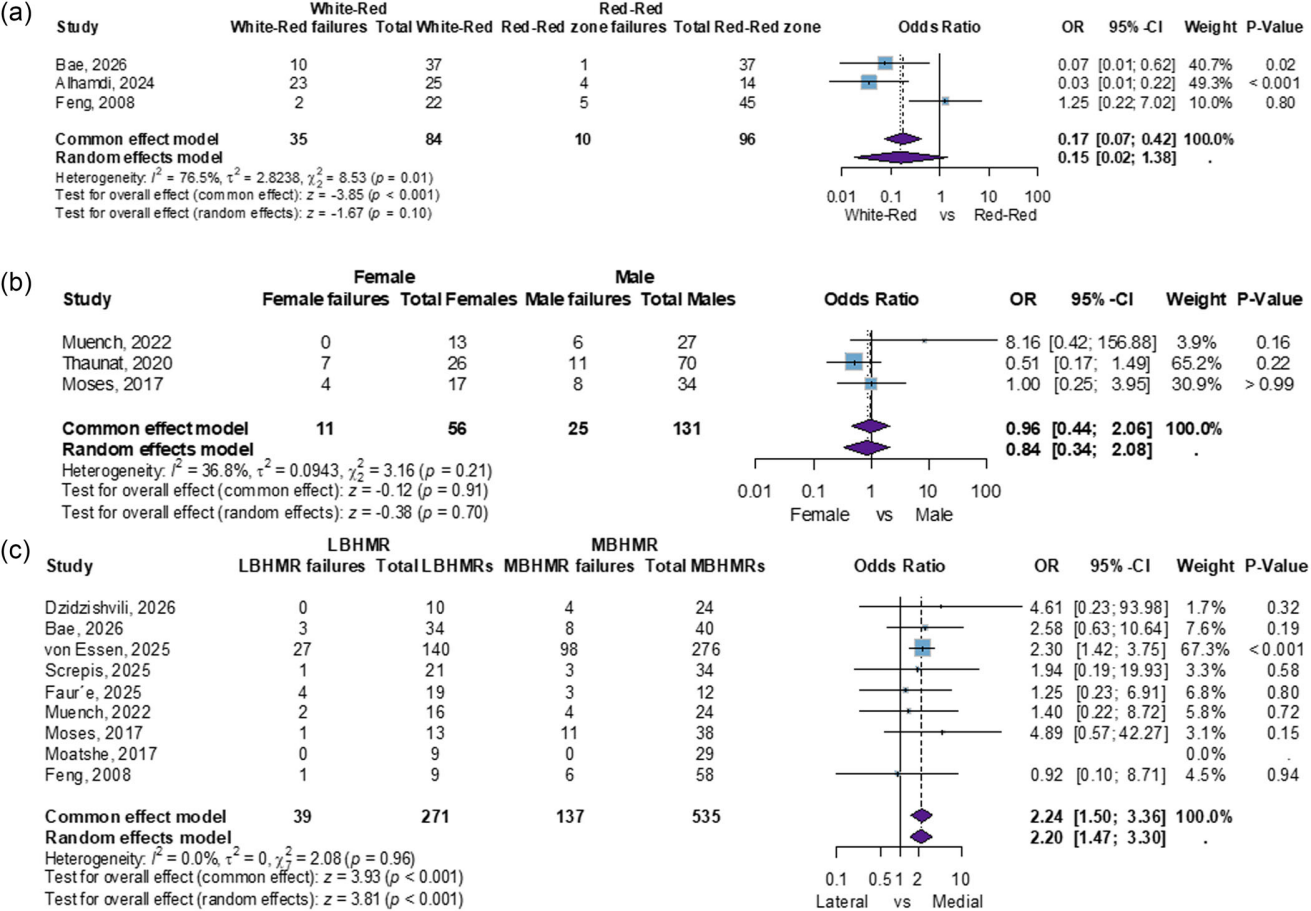
Forest plots of subgroup analyses evaluating factors associated with bucket‐handle meniscal repair (BHMR) failure. (a) Comparison of tears located in the white‐red versus red‐red vascular zones; (b) Comparison of failure rates between female and male patients; (c) Comparison of lateral (LBHMR) versus medial (MBHMR) repairs. CI, confidence interval; LBHMR, lateral bucket‐handle meniscal repair; MBHMR, medial bucket‐handle meniscal repair.

**Figure 5:**
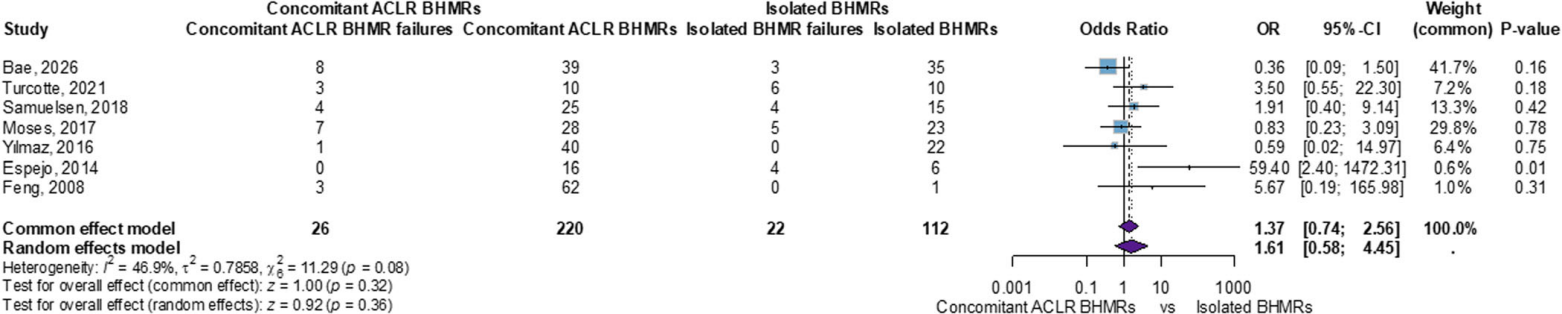
Forest plots comparing isolated BHMR versus Concomitant ACLR BHMR. ACLR, anterior cruciate ligament reconstruction; BHMR, bucket‐handle meniscal repair; CI, confidence interval.

Failure rates between BHMRs in the white‐red zone versus the red‐red zone indicated no significant difference (*p* = 0.10) under the random effect method (Figure 4a). The pooled odds ratio for failure in female patients relative to male patients was 0.96 (95% CI: 0.44–2.06, *p* = 0.91) (Figure 4b). As illustrated in Figure 4c, the pooled odds ratio for failure following medial BHMR relative to lateral BHMR was 2.20 (95% CI: 1.47–3.30, *p* < 0.001). Isolated BHMRs did not demonstrate a significantly increased odds of failure compared with concomitant ACLR BHMRs (OR = 1.37, 95% CI: 0.74–2.56, *p* = 0.32) (Figure 5).

### Meta‐regressions

As illustrated in Supporting Information: Figure S5, a significant positive association was identified between increasing follow‐up duration and reported failure rates (*β* = 0.12, 95% CI: 0.04–0.20, *p* < 0.01). No significant association was identified between increasing mean BMI and reported failure proportion (*β* = −0.17, 95% CI: −0.55 to 0.19, *p* = 0.35). Mean age, mean number of sutures, male sex, mean time to surgery (TTS), mean time to failure (TTF) and at the time of BHMR were not significantly associated with failure risk (all *p* > 0.05) (Supporting Information: Figures S5–S7).

### Publication bias assessment

Visual inspection of the funnel plot for pooled BHMR failure rate (Supporting Information: Figure S8) revealed notable asymmetry. Egger's linear regression test confirmed significant funnel plot asymmetry (*t* = −2.33, df = 30, *p* = 0.02). Trim‐and‐fill analysis identified six missing studies on the right side of the funnel plot.

### ROB assessment

The overall methodological quality of the evidence was moderate to good. Among case series, assessments using the JBI checklist demonstrated that most studies showed a low ROB in core domains, resulting in high overall quality (≥7 low‐risk domains) (Supporting Information: Figure S9). For cohort studies, ROBINS‐I evaluation indicated that most studies were at moderate to high ROB overall (Supporting Information: Figure S10). The most common concern was bias due to confounding, reflecting limited adjustment for baseline differences. Finally, RCTs, evaluated using the RoB 2.0 tool, were judged to have some concerns, primarily related to deviations from intended interventions and outcome reporting (Supporting Information: Figure S11). Supporting Information: Figures S3–S5 provide a summary of the ROB assessment across all included studies.

## DISCUSSION

The most notable finding of this study was that for this meta‐analysis of 32 studies encompassing over two decades of published evidence, BHMR demonstrated an overall pooled failure rate of approximately 21.5%. Failure rates increased progressively with longer follow‐up duration. Medial BHMRs were associated with more than a twofold higher odd of failure compared with lateral repairs. In contrast to several individual studies and the prior SRMA by Costa et al. [18], which reported significantly higher failure rates for isolated versus concomitant BHMRs with ACLR, the present analysis did not demonstrate a significant association for either comparison, this highlights the necessity of additional high‐quality studies to investigate this relationship more definitively.

Reported failure rates across individual studies varied widely, ranging from 0.0% [41, 58] to 69.2% [4], highlighting considerable variability in patient selection, surgical technique and follow‐up duration. Importantly, failure rates demonstrated a progressive increase with longer follow‐up, with meta‐regression confirming a significant positive association between follow‐up duration and failure risk. This finding suggests that shorter‐term studies may systematically underestimate true failure rates. These results are broadly consistent with, yet somewhat higher than, previously published systematic reviews. Costa et al. reported a pooled failure rate of 14.8% [18], while Tsakiri et al. similarly identified a pooled estimate of 14% [80], though the latter analysis included only five studies and was restricted to cohorts reporting Tegner activity scores. In contrast, Ardizzone et al. reported a substantially higher failure rate of 29.3%, although this was limited to repairs performed exclusively using the all‐inside technique [6]. This may be explained by device‐related issues. All‐inside meniscal repair devices generate significantly larger tissue penetrations compared with suture‐based techniques, potentially weakening the meniscus when multiple devices are placed.

Although this review did not demonstrate a significant association between the mean number of sutures and BHMR failure at the study level, the relationship between suture number and outcomes remains clinically nuanced. Hupperich et al. reported a strong association between increasing suture number and poorer clinical and healing outcomes, observing that the use of three or more sutures was associated with an increase in failure rate from 15.4% to 44% [33]. The authors suggested that a higher suture count may reflect either greater tear length (i.e., larger bucket‐handle lesions) or increased intraoperative difficulty in achieving stable reduction. These findings align with prior studies, which reported diminished healing rates in longer meniscal tears [8, 23, 33, 83].

Medial BHMR demonstrated a significantly higher risk of failure compared with lateral repairs, with more than a twofold increase in pooled odds of failure. Costa et al. similarly identified medial meniscus repair as a risk factor for failure [18]. This may be supported by biomechanical investigations demonstrating that the medial meniscus is subjected to greater contact pressures compared to the lateral meniscus [66, 87]. The medial meniscus exhibits reduced mobility relative to the lateral meniscus due to its firmer capsular and tibial attachments [41]. Arnoczky et al. and the more recent work by Crawford et al. indicate that peripheral vascularity is broadly comparable between the medial (up to 42%) and lateral (up to 48%) menisci [7, 19].

Interestingly, in our pooled subgroup analysis, failure rates between tears located in the red‐red zone and those in the white‐red zone did not demonstrate a significant difference. Modern repair techniques, biologic augmentation and variability in zone classification may partially overcome vascular limitations. Classic experimental and clinical studies have demonstrated that tears extending into the avascular central third of the meniscus are associated with markedly higher failure rates [13, 62, 64]. In contrast, the peripheral 25%–30% of meniscal tissue, corresponding to the ‘red‐red’ zone, exhibits superior healing potential [11, 31, 55, 57]. Foundational anatomic work by Arnoczky [7] and Cooper [17] established that vascular penetration in the adult meniscus is largely confined to the peripheral third. Age‐related vascular variation may also influence outcomes. While the adult meniscus demonstrates limited peripheral vascularity [16], children and adolescents may retain more extensive vascular penetration into inner zones, potentially explaining reports of successful repairs regardless of tear location in younger populations [8, 43, 45, 49, 62, 64].

Despite a higher pooled failure rate in isolated BHMRs (24.3% vs. 14.9%), isolated repair was not associated with significantly greater odds of failure compared with concomitant ACLR BHMRs. Although previous studies have suggested improved healing when meniscal repair is performed concomitantly with ACLR [13, 48, 63, 68, 84, 93], our analysis did not identify a statistically significant difference in failure rates between the two groups. This finding should be interpreted with caution, as more high‐quality studies are required to investigate this association.

Keyhani et al. [39] utilised a posterior arthroscopic approach and demonstrated no failures following BHMR, a finding that is consistent with the results reported by Ahn et al. [1]. The likelihood of chondral lesions and instrument damage may be decreased by using posteromedial ports [39, 50]. Additionally, this method eliminates the requirement for MCL needling or valgus stress force to provide extra space [12, 15]. They also applied vertical mattress sutures to improve meniscal healing through anatomical reduction [2, 15, 50]. However, further high‐quality comparative studies are necessary to determine whether the posterior approach independently improves repair outcomes.

This study has several limitations which must be acknowledged. First, the majority of included studies were observational, introducing inherent risks of selection bias, confounding and heterogeneity. Second, definitions of failure varied across studies, often combining clinical, radiological and reoperation criteria, which may have contributed to outcome heterogeneity. Third, meta‐regression was performed at the study level rather than the individual patient level, limiting causal inference. Additionally, incomplete reporting of certain variables restricted more granular analyses. Finally, although publication bias was formally assessed when feasible, the possibility of small‐study effects cannot be entirely excluded.

## CONCLUSION

BHMRs carry a substantial pooled failure rate of 21.5% that increases over time, with medial tears demonstrating significantly higher failure risks than lateral repairs. Notably, concomitant ACL reconstruction and tear vascularity (red‐red vs. white‐red) did not significantly alter failure odds. Given the observational nature and high heterogeneity of the literature, high‐quality prospective studies with standardised outcomes are urgently needed. Clinically, these findings emphasise long‐term surveillance and thorough patient counselling regarding re‐tear risks, particularly for medial repairs.

## AUTHOR CONTRIBUTIONS

Amirali Soleymani Nejad, Maryam Mousavi, Mehran Soleymanh performed the search strategy, study selection and screening. Data extraction performed by Amirali Soleymani nejad, Fardis Vosoughi and Mehran Soleymanha. Data analyses were performed by Mehran Soleymanh, Sohrab Keyhani and Luke V. Tollefson. Amirali Soleymani Nejad conducted the statistical calculations. Risk of bias assessment was conducted by Sohrab Keyhani, Amirali Soleymani Nejad, Mehran Soleymanh. Robert F. LaPrade, Mehran Soleymanha, Sohrab Keyhani and Luke V. Tollefson revised the manuscript. Robert F. LaPrade and Mehran Soleymanha supervised the work. All authors read and approved the final manuscript.

## CONFLICT OF INTEREST STATEMENT

The authors declare no conflicts of interest.

## ETHICS STATEMENT

Ethics approval was not required for this study because it is a systematic review and meta‐analysis based exclusively on data from previously published studies. No human participants or animals were directly involved, and no individual patient data were collected.

## Supporting information

Supporting File

## Data Availability

The data sets used and analysed during the current study are available from the corresponding author on reasonable request. The study protocol was prospectively registered in the PROSPERO international database (Registration ID: CRD420251272782).
