# Supplementary material for: Failure rates increase over time and are higher following medial arthroscopic bucket‐handle meniscal repair: A systematic review and meta‐analysis
Source: J Exp Orthop. 2026 Aug 27;13(3):e70854. doi: 10.1002/jeo2.70854 (PMC13521520; doi:10.1002/jeo2.70854)
Supplement: Supplementary file 1 [file JEO2-13-e70854-s001.docx]

**Supplementary Table S1. Database Search Strategies**

| Database | Search Strategy | Results |
| --- | --- | --- |
| PubMed | (("Menisci, Tibial"[MeSH] OR menisc*) AND ("displaced longitudinal tear" OR "bucket-handle" OR "bucket handle" OR buckethandle) AND ("Meniscal Repair"[MeSH] OR menisc* repair* OR meniscus sutur* OR "all-inside" OR "inside-out" OR "outside-in")) AND ("2005/01/01"[Date - Publication] : "3000"[Date - Publication]) | 264 |
| Scopus | TITLE-ABS-KEY ( ( (bucket-handle) OR (displaced longitudinal tear) OR (bucket handle) OR (buckethandle) ) AND ( (menisc* repair*) OR (menisc* sutur*) OR (all-inside) OR (inside-out) OR (outside-in) ) ) AND PUBYEAR > 2004 | 272 |
| Embase | ( 'bucket handle' OR buckethandle OR 'bucket handle tear'/exp OR 'bucket handle tear' OR 'displaced longitudinal tear') AND ( menisc* repair* OR 'meniscus repair'/exp OR 'meniscus repair' OR 'all-inside':ti,ab OR 'inside-out':ti,ab OR 'outside-in':ti,ab ) AND ( 'meniscus'/exp OR menisc* ) AND [2005-2026]/py | 298 |
| Web of Science | ALL= (((displaced longitudinal tear) OR (bucket-handle) OR (bucket handle) OR (buckethandle)) AND (menisc*) AND ((menisc* repair*) OR (menisc* sutur*) OR (all-inside) OR (inside-out) OR (outside-in))) | 301 |
| Cochrane Library | #1 MeSH descriptor Menisci, Tibial explode all trees  #2 menisc*  #3 #1 OR #2  #4 bucket handle  #5 buckethandle  #6 displaced longitudinal tear  #7 #4 OR #5 OR #6  #8 MeSH descriptor: Meniscal Repair explode all trees  #9 menisc* repair*  #10 menisc* sutur*  #11 all inside  #12 inside out  #13 outside in  #14 #8 OR #9 OR #10 OR #11 OR #12 OR #13  #15 #3 AND #7 AND #14 | 11 |

Supplementary Table S2. Summary of Data Clarifications Obtained Through Author Contact

| No. | Article title | Reason for Author Contact | Author Clarification |
| --- | --- | --- | --- |
| 1 | Impact of tear morphology and meniscal laterality on repair failure in stable knees: Medial repairs show over twofold risk | Clarification was sought regarding whether the reported time-to-failure values represented mean or median estimates | The authors confirmed that the reported values represent mean time to failure. |
| 2 | *Evaluation of Midterm Clinical and Radiographic Outcomes of Arthroscopically Repaired Vertical Longitudinal and Bucket-Handle Lateral Meniscal Tears* | Clarification was requested regarding the number of failures specifically attributable to bucket-handle meniscal repairs and whether an infection-related case should be considered a repair failure. | The authors confirmed that 2 of 21 bucket-handle meniscal repairs were classified as failures. The infection-related case was considered a postoperative complication and was not counted as a repair failure. |
| 3 | *Good mid- to long-term outcomes after meniscus bucket-handle tear repair: A comparative analysis with and without anterior cruciate ligament reconstruction* | Clarification was sought regarding whether the study cohort consisted exclusively of primary bucket-handle meniscal repairs or included revision repairs. | The authors confirmed that the cohort included both primary and revision repairs, comprising 42 primary repairs (76%) and 13 revision repairs (24%). |

**Figure S1.** World map depicting the geographic distribution of included studies reporting failure rates following bucket-handle meniscal repair (BHMR). Countries are shaded in blue according to the number of contributing publications, with darker shading representing higher research output.

**Figure S2.**  Forest plots depicting leave-one-out sensitivity analyses for the pooled failure proportion of bucket-handle meniscal repair (BHMR) stratified by study design. **(A)** Retrospective cohort studies (k = 24). **(B)** Case-series studies (k = 33). **Abbreviations:** BHMR, bucket-handle meniscal repair; CI, confidence interval; k, number of studies; I², Higgins' heterogeneity statistic.

**Figure S3.** Meta-analysis of failure rates following arthroscopic repair of bucket-handle meniscal tears. Forest plots show pooled failure proportions for (A) Medial bucket-handle meniscal repairs; and (B) Lateral bucket-handle meniscal repairs. Study-specific estimates are shown as squares with corresponding 95% confidence intervals, and pooled random-effects estimates are represented by diamonds.

**Figure S4.** Meta-analysis of failure rates following arthroscopic repair of bucket-handle meniscal tears. Forest plots show pooled failure proportions for (A) repairs performed concomitantly with ACL reconstruction and (B) isolated repairs. Study-specific estimates are shown as squares with corresponding 95% confidence intervals, and pooled random-effects estimates are represented by diamonds.

**Figure S5.** Meta-regression analyses of potential predictors of BHMR failure. (A) Mean length of follow-up; (B) Number of IO technique used.

**Figure S6.** Meta-regression bubble plots examining the association between study-level covariates and BHMR failure proportion. The logit-transformed failure rate is shown: (A) number of concomitant ACLR BHMRs; (B) number of isolated BHMRs; (C) number of AI technique used; (D) number of sutures used. The regression line suggests the direction and strength of the covariate-effect relationship.

**Figure S7.** Meta-regression bubble plots examining the effect of study-level characteristics on BHMR failure rates. Relationship between (A) number of males; (B) patient body mass index (BMI); (C) mean time to surgery; and (D) mean age with log-transformed BHMR failure rate.

**Figure S8.** Funnel plot assessing publication bias**.** The plot displays the distribution of included studies based on their effect sizes (logit-transformed proportions) and precision (standard error).

**Figure S9.** Traffic light plot of methodological quality assessment for included case-series studies using the JBI Critical Appraisal Checklist. Green represents low risk of bias, yellow represents unclear risk of bias, and red represents high risk of bias across the 10 assessed criteria. (A) Traffic-light plot of individual case-series studies; (B) Summary bar plot showing percentage distribution of risk-of-bias domains across case-series studies.

**Figure S10.** Risk of bias assessment of included studies. (A) Traffic-light plot of individual cohort studies using ROBINS-I; (B) Summary bar plot showing percentage distribution of risk-of-bias domains across cohort studies. Green represents low risk of bias, yellow represents unclear risk of bias, and red represents high risk of bias.

**Figure S11.** Risk of bias assessment. (A) Traffic light plot of RCTs using the RoB 2.0 tool; (B) Summary plot of risk of bias for RCTs. Green = low risk, yellow = unclear/some concerns, red = high risk.
